# Comparative molecular characterization of typical and exceptional responders in glioblastoma

**DOI:** 10.18632/oncotarget.25420

**Published:** 2018-06-19

**Authors:** Kristin Wipfler, Adam S. Cornish, Chittibabu Guda

**Affiliations:** ^1^ Department of Genetics, Cell Biology and Anatomy, University of Nebraska Medical Center, Omaha, NE 68198, USA; ^2^ Bioinformatics and Systems Biology Core, University of Nebraska Medical Center, Omaha, NE 68198, USA; ^3^ Department of Biochemistry and Molecular Biology, University of Nebraska Medical Center, Omaha, NE 68198, USA; ^4^ Fred and Pamela Buffett Cancer Center, University of Nebraska Medical Center, Omaha, NE 68198, USA

**Keywords:** glioblastoma, survival analysis, integrative analysis, exceptional responders, TCGA

## Abstract

Glioblastoma (GBM) is the most common and the deadliest type of primary brain tumor, with a median survival time of only 15 months despite aggressive treatment. Although most patients have an extremely poor prognosis, a relatively small number of patients survive far beyond the median survival time. Investigation of these exceptional responders has sparked a great deal of interest and is becoming an important focus in the field of cancer research. To investigate the molecular differences between typical and exceptional responders in GBM, comparative analyses of somatic mutations, copy number, methylation, and gene expression datasets from The Cancer Genome Atlas were performed, and the results of these analyses were integrated via gene ontology and pathway analyses to assess the functional significance of the differential aberrations. Less severe copy number loss of *CDKN2A*, lower expression of *CXCL8*, and *FLG* mutations are all associated with an exceptional response. Typical responders are characterized by upregulation of NF-κB signaling and of pro-inflammatory cytokines, while exceptional responders are characterized by upregulation of Alzheimer’s and Parkinson’s disease pathways as well as of genes involved in synaptic transmission. The upregulated pathways and processes in typical responders are consistently associated with more aggressive tumor phenotypes, while those in the exceptional responders suggest a retained ability in tumor cells to undergo cell death in response to treatment. With the upcoming launch of the National Cancer Institute’s Exceptional Responders Initiative, similar studies with much larger sample sizes will likely become possible, hopefully providing even more insight into the molecular differences between typical and exceptional responders.

## INTRODUCTION

Glioblastoma (GBM) is the most common and deadliest type of primary brain tumor [1]. It is highly malignant and nearly uniformly fatal, with a median survival time of only approximately 15 months despite aggressive treatment [2], including surgical resection followed by concurrent radiation and chemotherapy with temozolomide [1, 3]. Although most patients have an extremely poor outcome, a small number of patients survive far beyond the median survival time [4].

Recently, there has been a great deal of interest in investigating the molecular characteristics of exceptional responders in cancer [5]. Exceptional responders are patients who have a unique response to treatments that are not effective for most other patients. They typically achieve a complete or partial response that only up to 10% of patients experience, and they sustain that response for a much longer duration than the median response. The National Cancer Institute (NCI) is currently developing a new genomics database, the Exceptional Responders Initiative (ERI) to identify molecular features of these exceptional responders [6]. Several studies on this topic have already been published [5] which have helped uncover molecular alterations and mechanisms of resistance. With the recent shift of focus to exceptional responders in the field of cancer research, it is important to incorporate this concept into survival studies in cancer, particularly in cancers like GBM, in which most patients respond poorly to treatment but an exceptional few respond very positively.

In this study, we aim to analyze and integrate the results of somatic mutation, copy number variation, methylation, and gene expression analyses utilizing data from The Cancer Genome Atlas (TCGA) for typical and exceptional responders in GBM. These response groups were defined utilizing cutoff parameters specific to characteristics of GBM and guided by the concept of exceptional responders, with the goals of providing a clearer understanding of the molecular basis for these patients’ positive response to standard GBM therapy and revealing possible therapeutic targets or prognostic markers for GBM.

## RESULTS

### Sample selection and defining response groups

**Figure 1:**
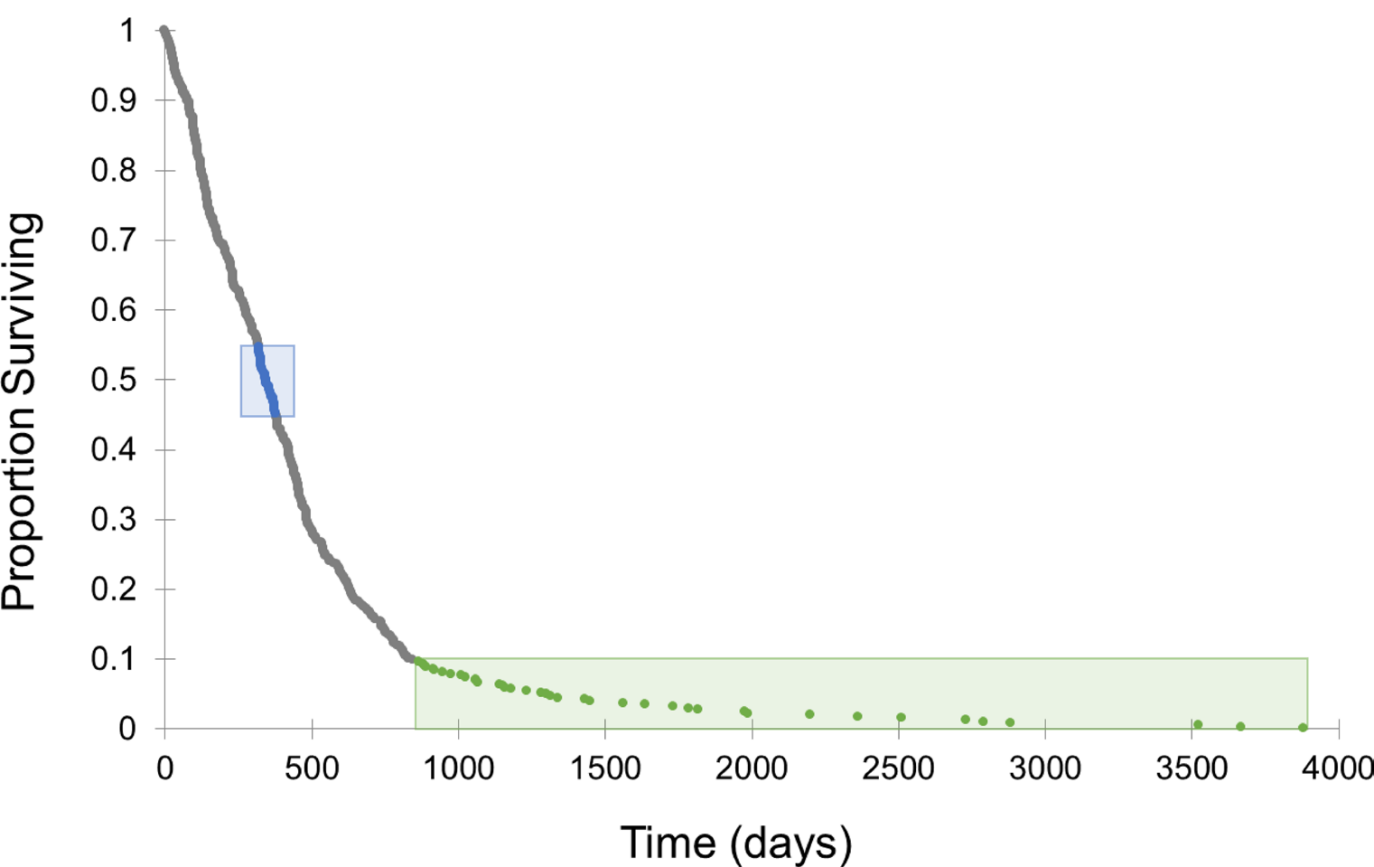
Survival curve for TCGA GBM dataset This curve includes the 408 TCGA GBM patients that met the inclusion criteria. The curve is characterized by a steep drop off centered around the median of 345 days, with a relatively small number of patients surviving beyond approximately 2.5 years. Typical responders are labeled in blue and exceptional responders are labeled in green.

After the application of the inclusion criteria to ensure that all patients in the analysis have known survival times and tumor samples taken prior to radiation and chemotherapy, 408 patients remained in the dataset. The Kaplan–Meier survival curve (Figure 1) for those 408 patients shows a steep drop in the first two years, with the survival time for the vast majority of patients within one year of the median 345 days, which is consistent with the median survival reported in the literature during the time period that most of the samples were obtained [7], primarily in the first decade of the 2000s. The curve levels off between two and three years, and a relatively small number of patients survive beyond that time. The patients within that range are in roughly the top 10% for survival time, which is the defining factor for the exceptional responders group.

After investigating potential confounding variables with linear regression models, age and sex were determined to be confounding. An age cutoff of ≥30 years was applied, which reduced the exceptional responders group by five patients and corrected for the confounding variable of age. Ethnicity was the same for all patients in this group (not Hispanic or Latino) and Karnofsky score, age, race, and diagnosis method were not significant predictors of survival. However, sex was predictive of outcome, with female patients enriched in the exceptional responders group (regression model *p =* 0.021, chi-squared test *p =* 0.034). Sex is only associated with survival in the typical and exceptional response groups, not in the full dataset of 408 patients, and was addressed in the methodology of each analysis.

The final dataset included 40 typical responders and 35 exceptional responders (Table 1, Supplementary Table 1). Males are more highly represented in the typical response group, and the exceptional responders tend to be younger with a mean age of 49.8 years compared to the typical responders’ mean age of 58.7 years. However, this age difference is not statistically significant. The median survival for the typical group is the same as the full dataset (345 days) with a range of 320-378 days. Median survival for the exceptional group is 1282 days (approximately 3.5 years) with a range of 864-3881 days (approximately 2.4–10.6 years).

**Table 1:** Descriptive statistics for the full dataset and response groups

	All	Typical responders	Exceptional responders
**Patients (*n*)**	408	40	35
Male	253	29	17
Female	155	11	18
**Age (years)**			
Mean	58.7	58.0	49.8
Range	10–89	33–73	30–74
**Survival (days)**			
Mean	459	347	1600
Median	345	345	1282
Range	3–3881	320–378	864–3881

Statistics on sample size, age, and survival time are included for the full group of patients that met the inclusion criteria as well as for the typical and exceptional response groups. Typical responders closely resemble normal characteristics for GBM in general, while exceptional responders tend to be younger (though this is not statistically significant) and have an equal representation of males and females as opposed to the usual higher proportion of males.

### Somatic mutations

Only one gene, *FLG* (filaggrin) was found to have a significantly different distribution of patients affected by somatic nonsynonymous mutations (chi-squared test *p =* 0.041). Just one typical responder (4.2%) harbored any somatic mutations in *FLG*, while six exceptional responders (25%) had one mutation each in *FLG* (Supplementary Table 2). All six of the mutations in the exceptional responders are missense variants that are predicted to have a moderate impact, and an equal number of males and females are affected.

### Copy number

Utilizing a log_2_ ratio cutoff of ±0.25 to define copy number gains/losses [8], several regions were identified as altered relative to normal (Table 2). Of the 1812 probes that meet the definition of copy number gain/loss, 1752 of them are losses, and 1201 of those have a greater magnitude in typical responders. Only 60 of these indicate copy number gains, and the magnitude is greater in typical responders for all 60 of them. Overall, 69.6% of the gains and losses have a larger magnitude in typical responders.

**Table 2:** Regions of copy number gain and loss

Gains	
Region and Gene(s)	Response Group Affected
7p11.2 *HPVC1*, VSTM2A^*^, LOC285878^*^, *SEC61G*, *EGFR*, *EFGR-AS1*, *LANCL2*, *VOPP1*, *FKBP9L*, *SEPT14*, *MRPS17*, *GBAS*, *PSPH*, *CCT6A*, *SNORA15*, *SUMF2*, *PHKG1*, *CHCHD2*, and *NUPR1L*	typical; exceptional to a lesser extent
7q21.2 *AKAP9*, *CYP51A1*, *LRRD1*, *KRIT1*, *ANKIB1*, *GATAD1*, *PEX1*, *RBM48*, *MGC16142*, *FAM133B*, and *CDK6*	typical
7q34 *PRSS3P2* and *PRSS2*	typical

Losses	
Region and Gene(s)	Response Group Affected
1q21.3 *LCE3C*	exceptional
8p11.22 *ADAM3A*	typical
9p21.3, 9p21.2 *FOCAD*, *MIR491*, *PTPLAD*, *IFNB1*, *IFNW1*, *IFNA21*, *IFNA4*, *IFNA7*, *IFNA10*, *IFNA16*, *IFNA17*, *IFNA14*, *IFNA22P*, *IFNA5*, *KLHL9*, *IFNA6*, *IFNA13*, *IFNA8*, *IFNA1*, *MIR31HG*, *IFNE*, *MIR31*, *MTAP*, CDKN2A-AS1^*^, *CDKN2A*, *CDKN2B-AS1*, *CDKN2B*, *DMRTA1*, *FLJ35282*, *ELAVL2*, *IZUMO3*, *TUSC1*, *LOC100506422*	typical; exceptional to a lesser extent
entirety of chromosome 10	typical and exceptional
11p15.4 *OR52N5*	typical
13q14.2 *DLEU2*, *MIR16-1*, *MIR15A*, *DLEU1*, and *ST13P4*	exceptional
15q11.2 OR4M2^*^ and OR4N4^*^	exceptional

Regions of copy number gain (mean log_2_ ratio > 0.25) and loss (mean log_2_ ratio < 0.25) are shown with lists of specific genes affected in each region. Genes in bold and labeled with a ^*^ reached statistical significance following multiple testing correction (*q <* 0.1) based on *t*-tests comparing typical and exceptional responders.

**Figure 2:**
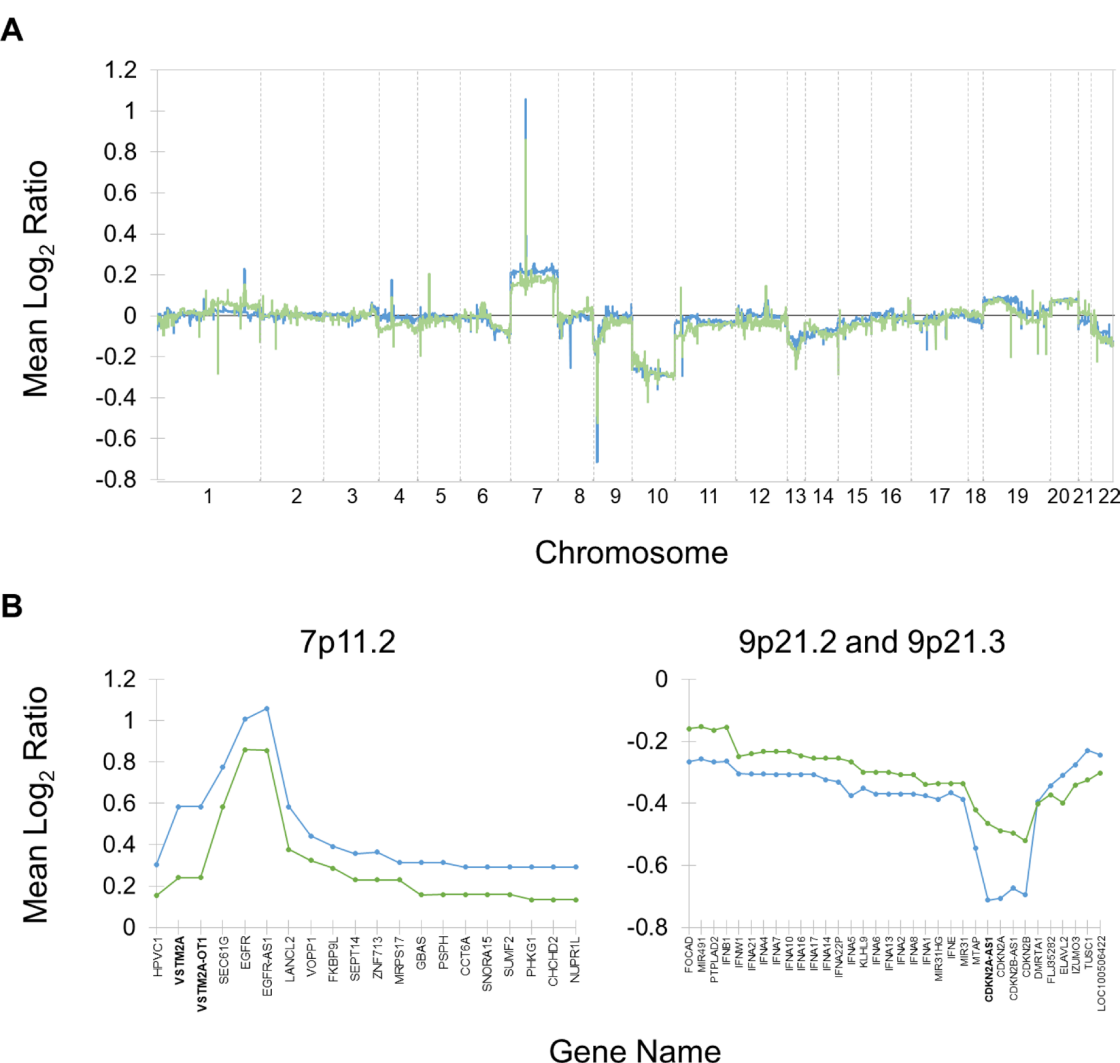
Copy number alterations in typical and exceptional responders relative to normal Mean log_2_ ratios for typical (blue) and exceptional (green) responders are shown. (**A**) Mean log_2_ ratios assessed at approximately 40,000 probes genome-wide, excluding sex chromosomes. The most prominent alterations are gains in chromosome 7 and losses in chromosome 9p and 10. Peaks tend to be of a greater magnitude in the typical response group. (**B**) Mean log_2_ ratios in regions that include differential copy number alterations between typical and exceptional responders. Both groups are characterized by gains in 7p11.2 and losses in 9p21.2 and 9p21.3, but in both cases the magnitude is greater in typical responders. Genes in bold (*VSTM2A*, *VSTM2A-OT1*, and *CDKN2A-AS1*) have differential mean log_2_ ratios that reach statistical significance.

The mean log_2_ ratios across the genome for each response group (Figure 2A) indicate that gains and losses in the same regions mostly occur consistently in both response groups, although the magnitude of the gain or loss typically appears to be larger in typical responders. Five genes were determined to be differentially altered between the response groups, including losses in *CDKN2A-AS1*, *OR4M2*, and *OR4N4* and gains in *VSTM2A* and *VSTM2A-OT1*. The sex-specific analyses did not yield any significant results. A more detailed look at two of the regions surrounding these significantly differentially altered genes (Figure 2B) confirms that the magnitude of gain or loss is greater in typical responders and reveals that several genes known to be associated with GBM [9] are immediately adjacent to some of the significantly differentially altered genes. Namely, *EGFR* (epidermal growth factor receptor) is adjacent to *VSTM2A* and *VSTM2A-OT1*, while *CDKN2A-AS1* overlaps with *CDKN2A* (cyclin dependent kinase inhibitor 2A) and is adjacent to *CDKN2B* (cyclin dependent kinase inhibitor 2B).

Due to their proximity to genes with significantly differentially altered copy numbers as well as their established connection to GBM [10], the rates of gains/amplifications (log_2_ ratio > 0.25/0.8) and losses/deletions (log_2_ ratio < 0.25/0.8) in *EGFR*, *CDKN2A*, and *CDKN2B* were investigated in the two response groups. While the copy number analysis described above was a genome-wide investigation of the continuous variable “log_2_ ratio,” this analysis focused on three genes of interest and was based on the categorical variables “gain/amplification” and “loss/deletion.” Typical responders were significantly more likely than exceptional responders to experience loss or deletion of *CDKN2A* (chi-squared test *p =* 0.038), but there was no significant difference between the response groups in the distribution of gains/amplifications of *EGFR* or losses/deletions of *CDKN2B*.

### Methylation

**Figure 3:**
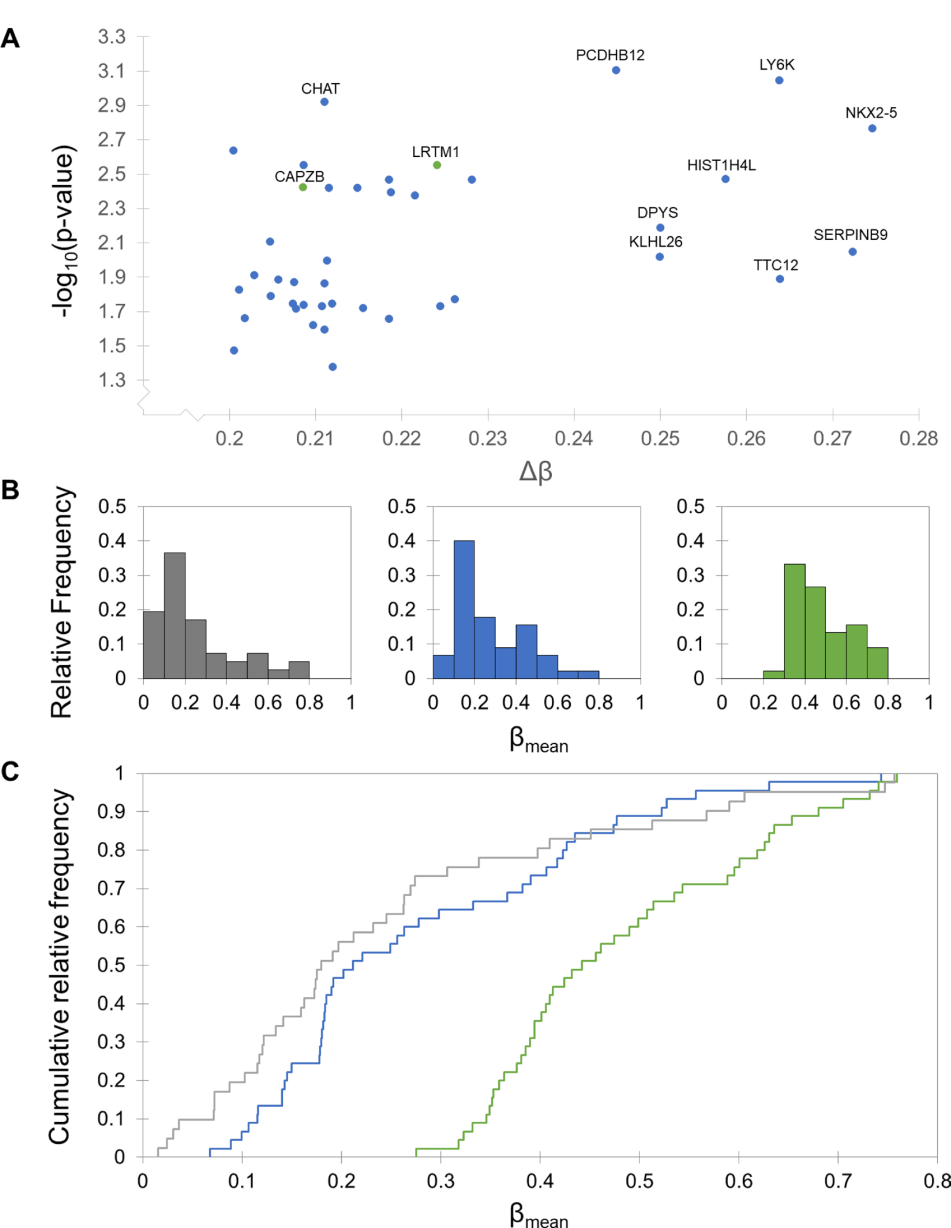
Differential methylation patterns between typical and exceptional responders Typical responders are shown in blue, exceptional in green, and normal in grey. (**A**) Modified volcano plot of significantly differentially methylated CpG sites between typical and exceptional responders. Each axis is skewed to reflect the cutoffs made to assess significance (*p* > 0.05 and ∆β > 0.2). Sites with a lower degree of methylation in typical responders are shown in blue and sites with a lower degree of methylation in exceptional responders are shown in green. Sites with the largest ∆β values and smallest *p* values are labeled with their associated gene name. (**B**) Histograms of β values in normal glial cells and each response group. The response group histograms include mean β values for the 45 CpG sites with ∆β values larger than 0.2, and the normal histogram includes β values for 41 of those sites (the remaining 4 sites were not assessed in the normal arrays). The distribution of β values in typical responders closely resembles the distribution for normal glial cells, while the exceptional responders are characterized by a shift towards larger β values. (**C**) Kolmogorov-Smirnov (KS) tests and cumulative distribution plots of β values. KS tests indicate that the distribution of β values for the CpG sites with ∆β > 0.2 is significantly different from both the typical and normal distributions (*p <* 0.0001). There is no difference between the distributions for the typical and normal groups. Cumulative distribution plots are shown for each of the three groups, indicating a clear shift in exceptional responders toward higher β values.

A total of 41 differentially methylated CpG sites corresponding to 37 unique genes were identified, 39 of which had a higher degree of methylation in the exceptional response group (Supplementary Table 3). A modified volcano plot (Figure 3A) indicates which of these sites are outliers with the highest ∆β values and the lowest *p* values. In the promoter analysis, a total of five differentially methylated promoters were identified, all with a higher degree of methylation in exceptional responders (Supplementary Table 4).

There are 45 CpG sites with a ∆β value greater than 0.2 between the typical and exceptional response groups, 41 of which are also present in the normal dataset (obtained from a 2014 study on methylation in neurons and glia [11]). Histograms for each of these groups (Figure 3B) show larger proportions of hypomethylated (β < 0.3 [12]) sites in the typical and normal groups and more moderate β values (0.3 < β < 0.7) in the exceptional group. This observation was investigated further with Kolmogorov-Smirnov (KS) tests and cumulative distribution plots for each group (Figure 3C). There is no difference in the distribution of β values between typical responders and normal glial cells (*p =* 0.127), but the exceptional response group β value distribution is significantly different from both of the other groups (*p <* 0.0001 in both cases). The D statistic, a measure of the magnitude of the difference between two datasets, is 0.622 for the typical versus exceptional comparison and 0.734 for the normal versus exceptional comparison.

### Gene expression

The analysis identified four significantly differentially expressed genes (Supplementary Table 5) between typical and exceptional responders. All four of them were identified in the full analysis and in each of the sex-specific analyses. *ETNPPL* (ethanolamine-phosphate phospho-lyase) and *SH3GL2* (SH3 domain containing GRB2 like 2, endophilin A1) were more highly expressed in exceptional responders, while *CXCL8* (interleukin 8) and *CCL20* (chemokine ligand 20) were more highly expressed in typical responders. Based on a known synergistic relationship between *CXCL8* and *CCL20* that promotes poor survival outcome in colorectal cancer [13], these two genes were further investigated as possible prognostic factors in GBM. Linear regression models built with the full TCGA GBM dataset indicate that *CXCL8* (but not *CCL20*) is predictive of survival time, with increased expression of *CXCL8* associated with reduced survival time. The overall model is statistically significant (*p <* 0.001), as is the *CXCL8* term (*p <* 0.001). The equation for the model is *survival time (days)* = *812–40.1* ^*^ *normalized signal of CXCL8*.

### Gene set enrichment analysis

Gene set enrichment analysis (GSEA) identified four gene sets enriched in exceptional responders and one gene set enriched in typical responders (Supplementary Table 6). The four gene sets enriched in exceptional responders (regulation of synaptic vesicle transport, regulation of neurotransmitter transport, positive regulation of calcium ion dependent exocytosis, and neurotransmitter secretion) are all associated with synapse function. The gene set enriched in typical responders was “negative regulation of cytokine biosynthetic process.” There were no significantly enriched gene sets identified when the analysis was divided by sex.

Manual inspection of the genes implicated by these enriched gene sets (Table 3) revealed several consistent trends in each response group. Multiple genes upregulated in typical responders were associated with the IL-17 and TNF signaling pathways. As expected based on the enriched gene sets, genes upregulated in exceptional responders were frequently associated with synaptic transmission and plasticity, but several were also associated with neurodegenerative diseases like Alzheimer’s and Parkinson’s.

**Table 3:** Significantly differentially altered genes across all analyses

	Typical responders		Exceptional responders	
**Copy number**	*VSTM2A* *LOC285878* *OR4M2* *OR4N4*		*CDKN2A-AS1* *CDKN2A*		
**Methylation**	*SLC15A3* *TTC12* *LRRC8E* *SUSD3* *LRRC61*				
**Gene expression**	*CXCL8* *CCL20*		*ETNPPL* *SH3GL2*		
**GSEA**	*LAG3* *SFTPD* *INHBA* *TRIB2* *KLF4* *NMI* *NFKB1* *INHBB* *IL6* *RNF128* *BCL3*		*NLGN1* *STXBP1* *DNM1* *PINK1* *SYNJ1* *CDK5* *SCAMP5* *CACNA1I* *SYT1* *KCNB1* *PLCB1* *CDK5R2* *NOS1* *CPM6B* *TOR1A* *CAMK2A*		*CACNA1A* *RIMS1* *KCNMB4* *SNCG* *MEF2C* *SNCAIP* *SYT11* *ACCN2* *NF1* *RAB3A* *RIMS3* *SNCA* *SYN1* *RAB5A* *RAB3GAP1* *PFN2*

The genes are separated by response group according to which group they are upregulated in. “Upregulated” for each data type is defined as: larger magnitude copy number gains/smaller magnitude copy number loss, lower promoter methylation, higher gene expression, and enrichment in GSEA.

### Gene ontology and pathway analyses

Utilizing the list of differentially altered genes identified in the copy number, methylation, and gene expression analyses in this study for each response group (Table 3), enriched gene ontology (GO) terms were identified in each response group. The GO term “cellular response to interleukin-1” was enriched in typical responders due to the presence of *CCL20* and *CXCL8*, while “intracellular transport” and “synaptic vesicle uncoating” were enriched in exceptional responders due to *CDKN2A* and *SH3GL2*.

KEGG pathway analyses performed with the same gene lists identified four pathways associated with the upregulated genes *CCL20* and *CXCL8* for typical responders, and no upregulated pathways in exceptional responders. The four KEGG pathways enriched in typical responders are the IL-17 signaling pathway, cytokine-cytokine interaction, chemokine signaling pathway, and rheumatoid arthritis.

## DISCUSSION

Although sex was a confounding variable for survival between the typical and exceptional response groups, it was not confounding in the full dataset. The incidence of GBM is higher in males, but sex has not been found to be predictive of prognosis or survival in GBM [14]. For these reasons, it seems likely that the identification of sex as a confounding variable occurred by chance for the selected response groups and is not actually predictive of survival. Although it is unlikely that sex is truly predictive of survival in GBM, the distribution of sexes between the two response groups was significantly skewed and had to be accounted for. When possible, this problem was addressed by performing sex-specific analyses in addition to the full analysis of typical versus exceptional responders, and then only results identified in all three analyses or only in the full analysis and not in the sex-specific analyses were included in the final results. When there was not a sufficient number of samples to perform sex-specific analyses, sex chromosomes were excluded from the analysis. These efforts seem to have been successful in controlling for the differential distribution of sex in the response groups. Prior to performing the methods to control for sex, many results were genes on the X or Y chromosome or were otherwise associated with sex. This was particularly true in the methylation analysis, in which 44% of significant sites and 57% of significant promoters were on the X chromosome prior to controlling for sex (data not shown). After the methods to control for the differential distribution of sexes were applied, the final results for all the data types were largely biologically relevant and not associated with any sex-specific biological processes.

The somatic mutation analysis indicated that exceptional responders are more likely to have nonsynonymous mutations in *FLG*. Overexpression of *FLG* is associated with low immune cell infiltration and early patient mortality in melanoma and ovarian cancer [15], while loss of function mutations in *FLG* are associated with lower cancer risk among some subpopulations [16]. This suggests that the high rate of nonsynonymous mutations in *FLG* in exceptional responders confers a prognostic benefit possibly related to immune cell infiltration.

Two of the significantly differentially altered genes identified in the copy number analysis, *OR4M2* and *OR4N4*, are olfactory receptors. There is huge variation in copy number among the general population in approximately 50% of olfactory receptors [17], so the appearance of these olfactory receptors in the significant results is likely an artifact and not actually associated with survival in GBM. The other significant copy number results led to the identification of the tumor suppressor [18] *CDKN2A* as a prognostic factor, with loss or deletion of this gene more likely to occur in typical responders than in exceptional responders.

A key finding of the methylation analysis was that among CpG sites that have a Δβ greater than 0.2, the typical group closely resembles the beta value distribution in normal glial cells, while the exceptional group is characterized by a higher level of methylation. Nearly all (95%) of the differentially methylated CpG sites and 100% of the differentially methylated promoters have a higher degree of methylation in exceptional responders. Histograms and cumulative distribution plots show a strong shift towards higher β values in exceptional responders, and this difference is statistically significant based on KS tests. This hypermethylation in exceptional responders relative to typical responders and normal glial cells suggests an increased level of transcriptional control that may confer a protective effect to exceptional responders.

Of the four genes that are differentially expressed between the response groups, *CXCL8*, *CCL20*, and *SH3GL2* have clear biological relevance. *CXCL8* is an angiogenic factor in many cancers [19, 20], *CCL20* promotes malignancy [21, 22], and together they synergize to promote a poor survival outcome via induction of epithelial-mesenchymal transition in colorectal cancer [13]. Both of those genes were overexpressed in typical responders relative to exceptional responders, and that is likely contributing to a poorer prognosis. *CXCL8* in particular is significantly predictive of survival time, and the linear regression model suggests that for every one unit increase in *CXCL8* expression, there is an associated 40-day reduction in survival. *SH3GL2* is a positive prognostic factor in head and neck squamous cell carcinoma [23] and is more highly expressed in exceptional responders. *SH3GL2* is targeted by mir330, which promotes malignancy in glioma cell lines, suggesting that reduced expression of *SH3GL2* results in more aggressive tumors [24]. The overexpression of *SH3GL2* in exceptional responders is consistent with a more positive prognosis.

Six of the genes contributing to the enriched gene sets in exceptional responders (*STXBP1*, *DNM1*, *SYNJ1*, *KCNB1*, *PLCB1*, and *CACNA1A*) are among a group of genes that have been implicated in early infantile epileptic encephalopathy (EIEE) [25], an extremely debilitating disorder characterized by uncontrollable seizures and severe mental retardation [26]. Mutations in these genes are associated with EIEE, but it appears that overexpression of these genes is associated with a positive prognosis in GBM.

The results from several analyses suggest that typical responders are characterized by aberrations of a greater magnitude than exceptional responders. The copy number analysis revealed that while most copy number alterations are consistent across both response groups, the magnitude is consistently larger in the typical responders. Methylation levels at CpG sites with a large degree of variation between the response groups are almost invariably lower in typical responders, suggesting that exceptional responders have increased gene silencing and transcriptional control. The somewhat lower level of disorder in exceptional responders may be contributing to their better prognosis.

Several pathways and biological processes were implicated in one response group or the other throughout this study. These include the upregulation of NF-κB and cytokine signaling in typical responders and the upregulation of key players in synaptic plasticity and neurodegenerative diseases in exceptional responders.

NF-κB was determined to be upregulated in typical responders relative to exceptional responders. *NFKB1*, a key player in the NF-κB pathway [27], was implicated in the GSEA analysis. Several other genes associated with NF-κB signaling were present in the significant results for typical responders, including *IL6*, *BCL3*, and *TRIB2*. Activation of NF-κB in GBM has been shown to contribute to angiogenesis and temozolomide resistance [29]. Enrichment of this pathway in typical responders may be partially responsible for their worse prognosis.

Cytokine signaling was upregulated in typical responders as well. Several pro-inflammatory cytokines and their associated pathways, which are known to promote cancer cell proliferation in many cases [30], were implicated in typical responders throughout this study, including *IL6*, *CCL20*, *CXCL8*, TNF signaling, and IL-17 signaling. Both of those signaling pathways are associated with more aggressive tumor phenotypes when they are upregulated [28, 31]. *CXCL8* and *CCL20* are perhaps the most prominent of these results, as they were the only two genes with significantly higher expression in typical responders compared to exceptional, and they are the two genes responsible for the only enriched GO term and all of the enriched KEGG pathways in typical responders. *CXCL8* was also found to be predictive of survival outcome in the full TCGA GBM dataset, with increased expression associated with significantly shorter survival times. Typical responders are characterized by upregulation of pro-inflammatory cytokine signaling, and exceptional responders do not share this trait. This lack of pro-inflammatory signaling in exceptional responders may result in a better prognosis.

One of the enriched GO terms and all the GSEA gene sets enriched in exceptional responders are directly related to synaptic transmission and synaptic plasticity. Autophagy pathways and synaptic plasticity pathways have a lot of overlap, and it has been proposed that autophagy plays a direct role in synaptic plasticity [32, 33]. Perhaps the upregulation of genes related to synaptic transmission in exceptional responders leaves their tumor cells more susceptible to autophagic cell death. Glioblastoma cells are more likely to respond to autophagy-inducing therapies than to apoptosis-inducing therapies [34], and it is possible that this characteristic of exceptional responders increases this positive response even further. It may also be the case that some other aspect of synaptic transmission and synaptic plasticity confers a benefit to exceptional responders.

Both Alzheimer’s disease and Parkinson’s disease pathways were implicated in exceptional responders based on the GSEA results. Both of these diseases are characterized by cell death through autophagy and/or apoptosis [35, 36]. The implication of Parkinson’s disease is of particular interest, as several epidemiological studies indicate an inverse association between cancer risk and Parkinson’s disease [37]. While activation of these pathways is certainly detrimental in neurodegenerative diseases, in cancer it may result in increased sensitivity to treatment. Tumors of exceptional responders with upregulation of these pathways may retain the ability to undergo cell death in response to treatment, granting exceptional responders a better prognosis.

The results generated in this study have provided some insight into the molecular differences between typical and exceptional responders in GBM. Upregulated pathways and processes in typical responders are consistently associated with more aggressive tumor phenotypes that may be partly responsible for the poor response to treatment that most GBM patients exhibit. Upregulated pathways and processes in exceptional responders may indicate that the small number of patients who respond very well to treatment have retained the ability to undergo cell death via autophagy in response to treatment. As the interest in exceptional responders grows, and with the upcoming launch of the ERI, similar studies with much larger sample sizes will likely be performed, which will provide even more insight into the molecular differences between exceptional and typical responders. Ideally this will ultimately lead to improved treatments and more positive outcomes for GBM patients.

## MATERIALS AND METHODS

### Sample selection and defining response groups

Inclusion criteria were applied utilizing clinical information contained within the TCGA Biotab files for GBM. Only untreated samples from patients with known survival times were included in the survival analysis to ensure that no samples in the study had been exposed to radiation or other treatments that may influence results. Only primary GBM samples were included in order to avoid statistical noise from secondary GBM samples. The top 10% of patients with the longest survival times were designated as “exceptional responders” based on the Kaplan–Meier survival curve, and the median 10% of patients were classified as “typical responders” so that the sample sizes matched. Linear regression models were generated to investigate possible confounding variables that may influence survival, including sex, race, ethnicity, diagnosis method, age, and Karnofsky score.

### Statistics

Linear regression models were created with XLSTAT. The term with the highest non-significant *p*-value was removed and the model was regenerated until the overall model and each term were significant (*p <* 0.05). All *t*-tests performed were Welch’s unequal variances *t*-tests and multiple testing was performed using the Benjamini-Hochberg false discovery rate (FDR) method [38] (*q <* 0.1 in all cases except for the gene set enrichment analysis, which was set to the default of *q <* 0.25). Chi-squared tests were performed when comparing categorical variables (*p <* 0.05). Kolmogorov-Smirnov (KS) tests were performed to compare distributions.

### Somatic mutations

Variant Call Format (VCF) files were acquired from the GDC on April 10, 2017 for 24 patients in each response group using curl (v. 7.52.1) via the GDC Application Programming Interface (API) using “data_type” = “Raw Simple Somatic Mutation”, workflow_type = “MuSE”, and sample_type = “Primary Tumor” as filters. Following this, the VCF files were annotated using snpEff (v. 4.3k) and human genome version GRCh38.d1.v1 which is the same genome used by the TCGA GBM project to identify variants in these samples. To assess the number of impactful mutations found per group, six classes of variations were used: loss of a start codon (coded as “start_lost” by snpEff), loss of a stop codon (stop_lost), gain of a stop codon (stop_gained), modification of a splice acceptor site (splice_acceptor), modification of a splice donor site (splice_donor), and missense mutations (missense). Using custom scripts, the mutations were then pooled such that, if a gene were mutated in a sample, it would only be counted once, even in the rare cases where, in a single patient, a gene had multiple mutations. The number of genes mutated per group was calculated and a cutoff was applied in which a gene had to have been mutated in at least three patients in one group to be worth further consideration.

### Copy number

Affymetrix Genome-Wide Human SNP Array 6.0 CEL files were obtained from the GDC Legacy Archive on April 15, 2017 for 38 typical responders and 34 exceptional responders. The files were divided into four groups based on response group and sex, and they were processed with the R tool Rawcopy [39]. Log_2_ ratio values (relative to normal) obtained from the genelist files generated by Rawcopy were compared between typical and exceptional responders to identify any differential gains or losses. A log_2_ ratio cutoff of ± 0.25 was used to define a copy number gain/loss and a cutoff of ± 0.8 was used to define amplification/deletion [8]. Only probes where the mean log_2_ ratio indicated a gain or loss for at least one of the response groups were included in the analysis. An additional cutoff was applied in which the difference in the mean log_2_ ratio between typical and exceptional responders must be > 0.2. Redundant probes (probes for the same gene with the same log_2_ ratio value) were removed. Following the full analysis, sex-specific analyses were performed with sex chromosomes excluded.

### Methylation

Illumina HumanMethylation27 idat files were acquired from the GDC Legacy Archive on September 27, 2016 for 16 typical responders and 16 exceptional responders. The analysis was performed with the R package RnBeads [40]. The arrays were normalized with the beta-mixture quantile normalization method and the Greedycut algorithm was utilized for filtering. There was not an adequate number of samples to perform sex-specific analysis, so sex chromosomes were excluded from the analysis. The resulting lists of CpG sites and promoters were narrowed further with a ∆β (the absolute value of the difference between mean β values for typical and exceptional responders) cutoff of 0.2 [41–43]. The “normal” dataset was obtained from a 2013 study in which Illumina HumanMethylation450 experiments were performed for glial cells of six different subjects, with two experiments for each subject [11]. Mean β values were calculated from the signal intensities for all 12 sets.

### Gene expression

Affymetrix HT Human Genome U133 DNA microarray CEL files were obtained from the GDC Legacy Archive on August 5, 2016 for 33 exceptional responders and 34 typical responders. The arrays were normalized with GCRMA [44] and filtering was performed using the nsFilter function of the genefilter package in R [45]. Quality control tests were performed, including boxplots of probe intensities and density versus intensity histograms, before and after normalization. Differential expression analysis was performed with limma [46]. A log_2_ fold change cutoff of ± 1.5 was applied to the resulting list of probes. The analysis was performed on the full dataset and also for males and females separately. Only genes implicated in all three analyses or in the full analysis but not the sex-specific analyses were considered to be significantly differentially expressed between the two response groups. The prognostic value of *CCL20* and *CXCL8* was investigated with linear regression models on the full TCGA GBM dataset that met the inclusion criteria (385 patients).

### Gene set enrichment analysis

Using the same CEL files from the gene expression analysis, version 2.2.4 of the Broad Institute’s Gene Set Enrichment Analysis (GSEA) tool [47] was used to detect enrichment of gene sets between exceptional and typical responders as well as male and female patients. The CEL files were converted to Gene Count files using the ExpressionFileCreator module found in GenePattern. Normalization was performed with GCRMA in conjunction with quantile normalization. GSEA was run using the c5.all.v6 database, with 1000 permutations performed using “phenotype” as the permutation type.

### Gene ontology and pathway analyses

Differentially expressed or methylated genes and genes with differential copy number gains/losses were combined and used as the input for GO and KEGG pathway analyses [48] for each response group. The GO analysis was performed using the Cytoscape plug-in ClueGO [49] with all four GO types selected, GO Term Fusion enabled, and results restricted to pathways with *p <* 0.05 after Benjamini-Hochberg FDR multiple testing correction.

## SUPPLEMENTARY MATERIALS TABLES



## Abbreviations

GBM: Glioblastoma
NCI: National Cancer Institute
ERI: Exceptional Responders Initiative
TCGA: The Cancer Genome Atlas
*FLG*: filaggrin
*EGFR*: epidermal growth factor receptor
*CDKN2A/B*: cyclin dependent kinase inhibitor 2A/B
FDR: false discovery rate
KS: Kolmogorov-Smirnov
*ETNPPL*: ethanolamine-phosphate phospho-lyase
*SH3GL2*: SH3 domain containing GRB2 like 2, endophilin A1
*CXCL8*: interleukin 8
*CCL20*: chemokine ligand 20
GSEA: gene set enrichment analysis
GO: gene ontology
KEGG: Kyoto Encyclopedia of Genes and Genomes
EIEE: early infantile epileptic encephalopathy
