# Supplementary material for: Comparative molecular characterization of typical and exceptional responders in glioblastoma
Source: Oncotarget. 2018 Jun 19;9(47):28421–33. doi: 10.18632/oncotarget.25420 (PMC6033343; doi:10.18632/oncotarget.25420)
Supplement: Supplementary file 1 [file oncotarget-09-28421-s001.pdf]

Comparative molecular characterization of typical and exceptional responders in glioblastoma

SUPPLEMENTARY MATERIALS

Supplementary Table 1: Sample number by sex and response group for each data type

	Typical responders	Exceptional responders
**Somatic mutations**		
Male	20	9
Female	4	15
**Copy number**		
Male	27	17
Female	11	17
**Methylation**		
Male	10	6
Female	3	13
**Gene expression**		
Male	23	16
Female	11	17

Supplementary Table 2: Genes with four or more patients affected by somatic mutations in at least one response group

Gene	*n* (typical)	*n* (exceptional)
TP53	8	9
EGFR	8	7
TTN	4	8
MUC16	8	3
PTEN	-	7
RYR2	3	6
FLG	-	6
SYNE1	5	3
LPA	5	-
MUC3A	3	4
NF1	3	4
CDH9	-	4
CKMT1A	4	-
FILIP1	4	-
KIAA1210	4	-
MGAM	-	4
MROH2B	4	-
MUC4	-	4
NXF3	4	-
OGT	-	4
PACS1	4	-
PHLDB2	-	4
PIK3CA	4	-
PKHD1	4	-
PLB1	4	-
PLXNA2	4	-
RAD51AP2	4	-
RELN	4	-
RYR3	4	-
SEC63	4	-
SLIT3	4	-
UMODL1	-	4
XIRP2	4	-

N indicates the number of patients with at least one somatic mutation identified in the associated gene. If fewer than three patients were affected, *N* is not listed.

Supplementary Table 3: Significantly differentially methylated CpG sites

cgid	gene name	mean β exceptional	mean β typical	Δβ	*p* value
cg12343638	*PCDHB12*	0.508	0.263	0.245	0.001
cg08569678	*LY6K*	0.442	0.178	0.264	0.001
cg12052765	*CHAT*	0.351	0.140	0.211	0.001
cg03294619	*NKX2-5*	0.364	0.089	0.275	0.002
cg21480743	*PTEN*	0.381	0.180	0.201	0.002
cg04369341	*C20orf100*	0.635	0.427	0.209	0.003
cg11532513	*LRTM1*	0.406	0.630	0.224	0.003
cg17651821	*HIST1H4L*	0.514	0.256	0.258	0.003
cg25957124	*DNAH3*	0.705	0.477	0.228	0.003
cg01888566	*MEST*	0.741	0.522	0.219	0.003
cg23519022	*CAPZB*	0.535	0.743	0.209	0.004
cg24101578	*CDH22*	0.618	0.407	0.212	0.004
cg25946389	*MGMT*	0.395	0.180	0.215	0.004
cg25509184	*CFTR*	0.601	0.382	0.219	0.004
cg09522147	*KRT7*	0.589	0.367	0.222	0.004
cg10303487	*DPYS*	0.432	0.182	0.250	0.007
cg09595479	*PRPH*	0.732	0.527	0.205	0.008
cg18676237	*SERPINB9*	0.474	0.202	0.272	0.009
cg12558519	*KLHL26*	0.349	0.099	0.250	0.010
cg26980692	*SLC15A3*	0.401	0.190	0.211	0.010
cg00949442	*ABCA3*	0.424	0.221	0.203	0.012
cg24264506	*TTC12*	0.456	0.192	0.264	0.013
cg16363586	*BST2*	0.680	0.474	0.206	0.013
cg27090216	*TNFRSF10C*	0.323	0.116	0.208	0.014
cg13067215	*CGI-38*	0.318	0.107	0.211	0.014
cg20050826	*K6IRS2*	0.412	0.211	0.201	0.015
cg21215336	*LRRC8E*	0.595	0.391	0.205	0.016
cg12981137	*MGMT*	0.376	0.150	0.226	0.017
cg01009664	*TRH*	0.461	0.249	0.212	0.018
cg09160477	*SUSD3*	0.275	0.067	0.207	0.018
cg07753583	*LRRC61*	0.626	0.417	0.209	0.018
cg07952391	*FLJ10916*	0.409	0.185	0.225	0.019
cg23244913	*HCG9*	0.543	0.333	0.211	0.019
cg12177743	*TTC12*	0.332	0.116	0.216	0.019
cg06274159	*ZFP42*	0.631	0.423	0.208	0.019
cg25057743	*PTHR2*	0.385	0.183	0.202	0.022
cg07260592	*LPA*	0.654	0.435	0.219	0.022
cg17965019	*HIST1H3J*	0.353	0.143	0.210	0.024
cg17860158	*CNTN2*	0.394	0.183	0.211	0.026
cg12768605	*LYPD5*	0.499	0.298	0.201	0.034
cg00630164	*KCNQ4*	0.489	0.277	0.212	0.042

These 41 CpG sites were determined to be differentially methylated between typical and exceptional responders following a cutoff of 0.2 and multiple testing correction ($q < 0.1$). The raw *p* values are shown.

Supplementary Table 4: Significantly differentially methylated promoters

gene name	mean β exceptional	mean β typical	Δβ	combined *p* value
SLC15A3	0.401	0.190	0.211	0.010
TTC12	0.394	0.154	0.240	0.012
LRRC8E	0.595	0.391	0.205	0.016
SUSD3	0.275	0.067	0.207	0.018
LRRC61	0.626	0.417	0.209	0.018

The promoters of these five genes were determined to be differentially methylated between typical and exceptional responders following a cutoff of 0.2 and multiple testing correction ($q < 0.1$) based on the degree of methylation of all the CpG sites that fall within the promoter range (1.5 kb upstream and 0.5 kb downstream of the transcription start site) for each gene. The raw combined *p* values are shown.

Supplementary Table 5: Significantly differentially expressed genes

Gene	$\log_2$FC (all)	$\log_2$FC (female)	$\log_2$FC (male)	*p* value (all)	*p* value (female)	*p* value (male)
ETNPPL	1.553	1.931	1.659	0.023	0.070	0.056
SH3GL2	1.771	2.301	1.758	0.002	0.009	0.021
CXCL8	–1.735<br>–1.863	–3.094<br>–2.345	–1.904	0.004<br>0.005	0.001<br>0.013	0.033
CCL20	–1.751	–2.383	-1.520	0.004	0.016	0.049

Four genes were determined to be significantly differentially expressed between typical and exceptional responders following multiple testing correction ($q < 0.1$) and comparison to the sex-specific analyses. *CXCL8* had two significant probes. Log_2 fold change and *p* values are shown for the full analysis and for each sex-specific analysis. *ETNPPL* and *SH3GL2* are more highly expressed in exceptional responders and *CXCL8* and *CCL20* are more highly expressed in typical responders.

Supplementary Table 6: Enriched gene sets identified by GSEA

Name	Enriched in	Normalized enrichment score	*p* value	FDR *q* value
regulation of synaptic vesicle transport	exceptional	2.062	$p < 0.001$	0.124
regulation of neurotransmitter transport	exceptional	2.025	$p < 0.001$	0.113
positive regulation of calcium ion dependent exocytosis	exceptional	2.008	$p < 0.001$	0.109
neurotransmitter secretion	exceptional	2.006	$p < 0.001$	0.078
negative regulation of cytokine biosynthetic process	typical	–2.068	$p < 0.001$	0.154

GSEA identified five enriched gene sets, four of which are enriched in exceptional responders.
